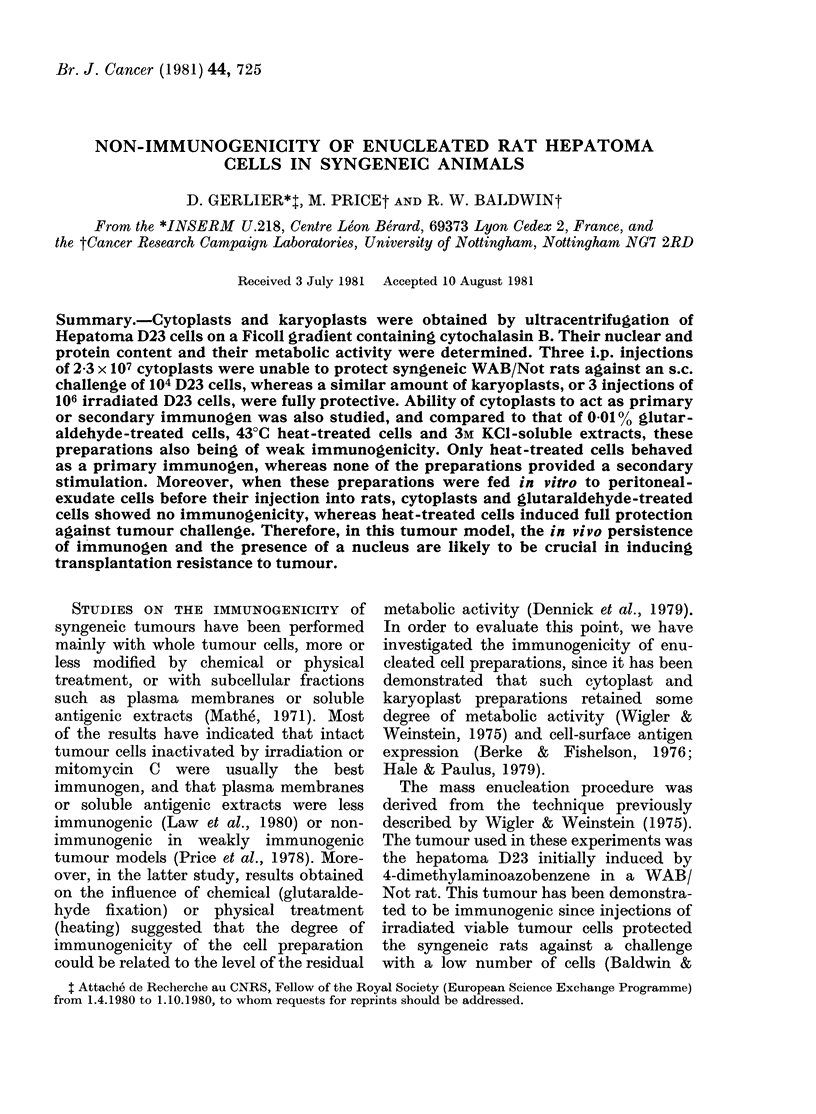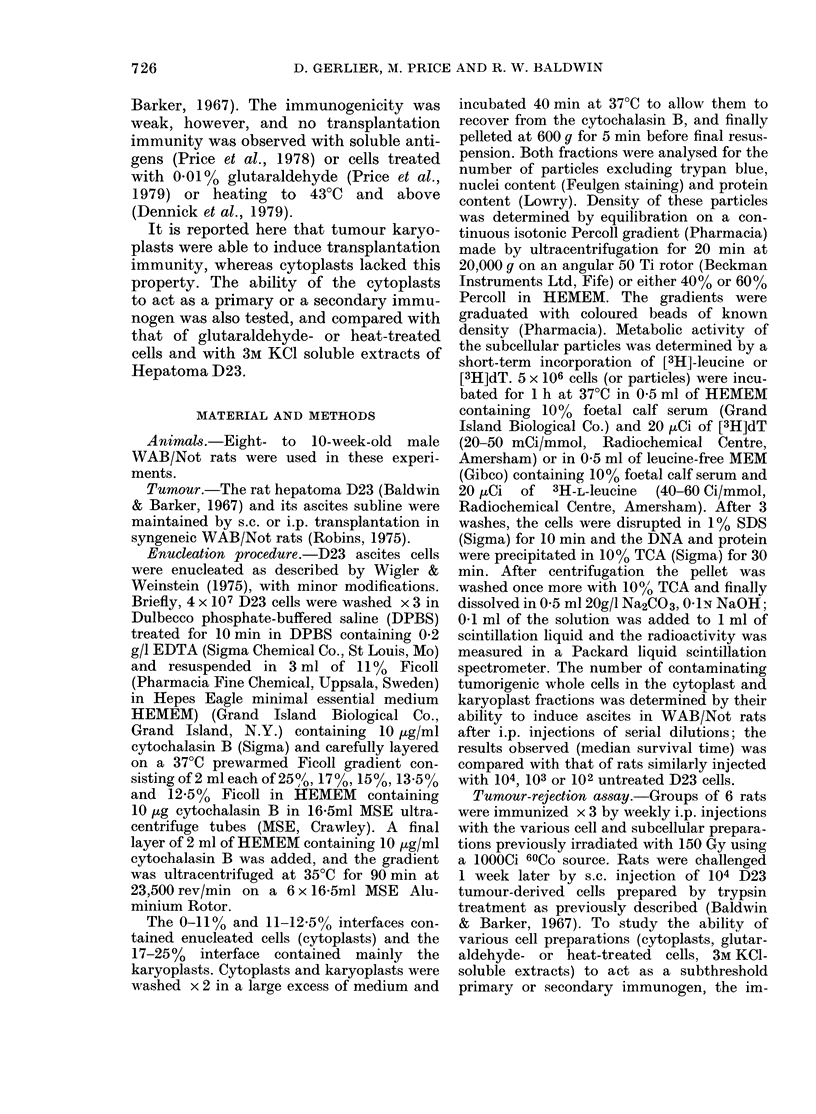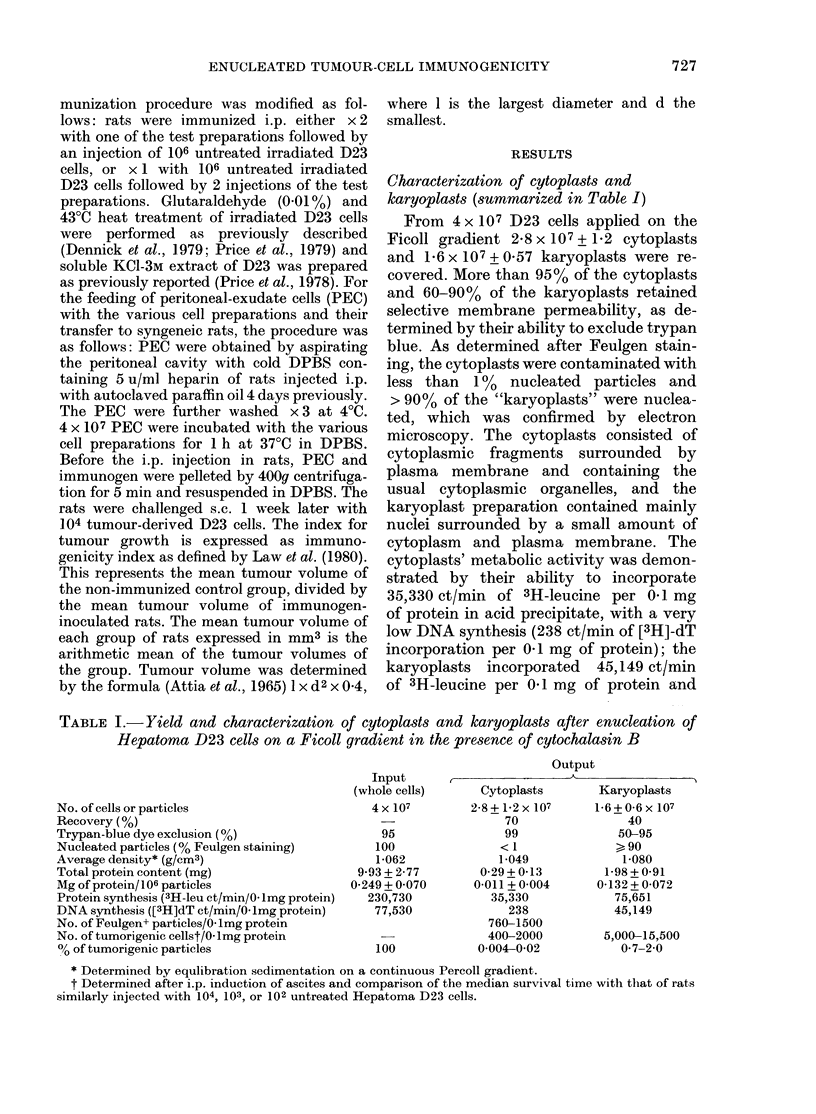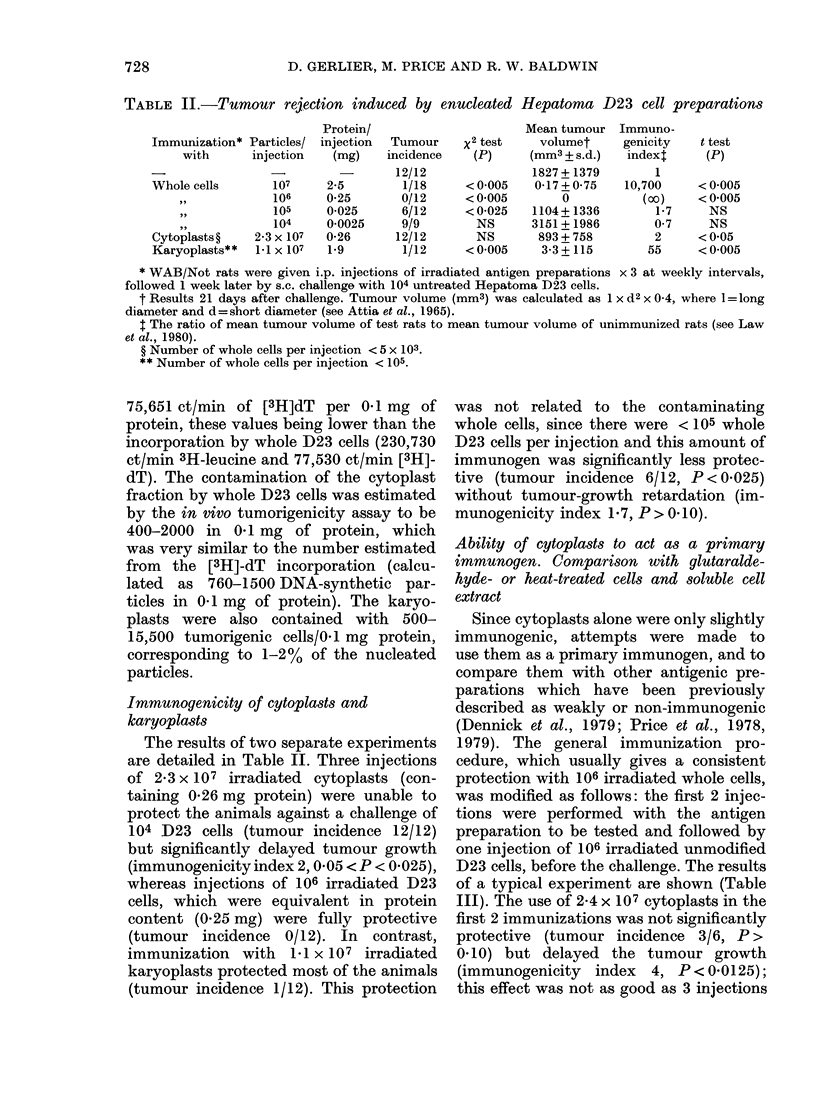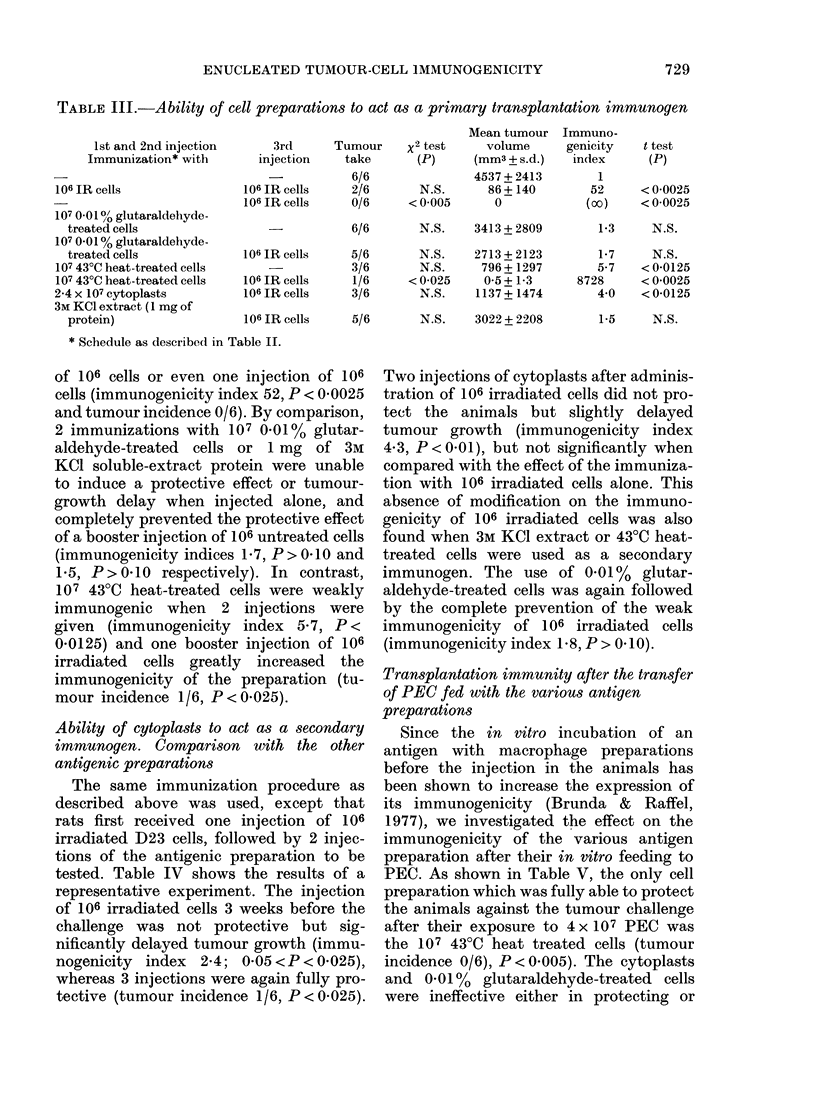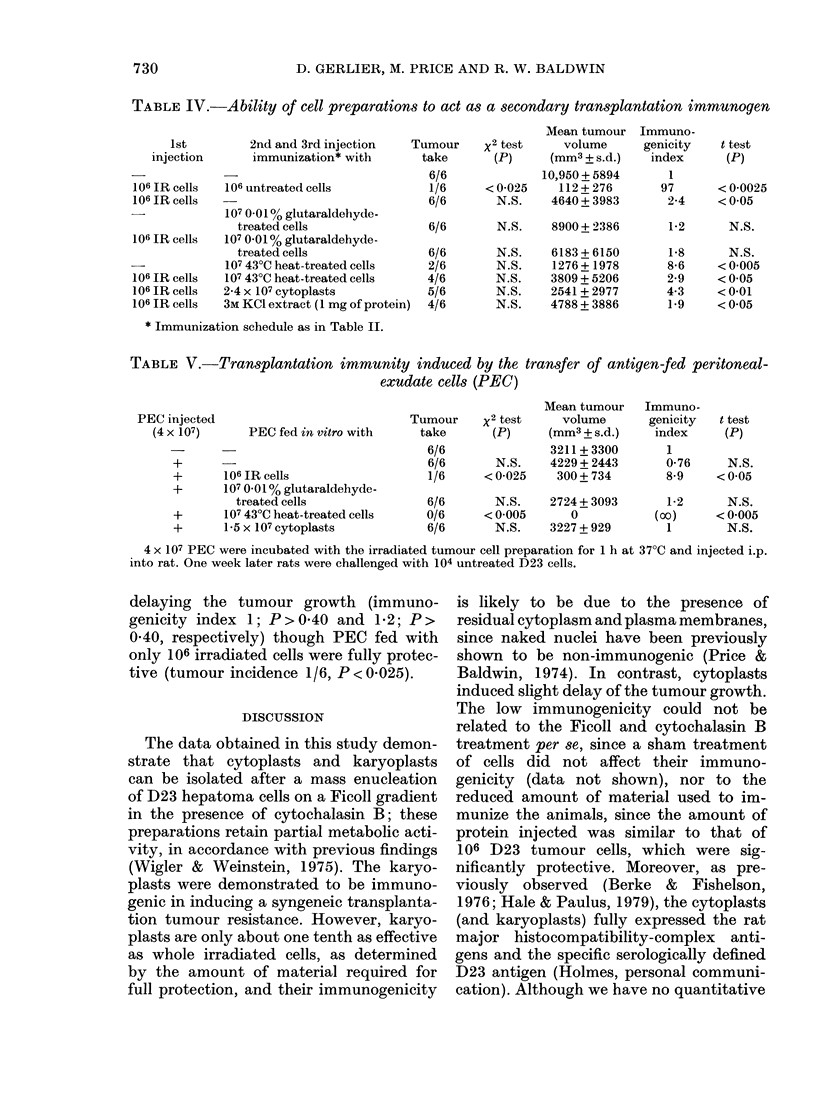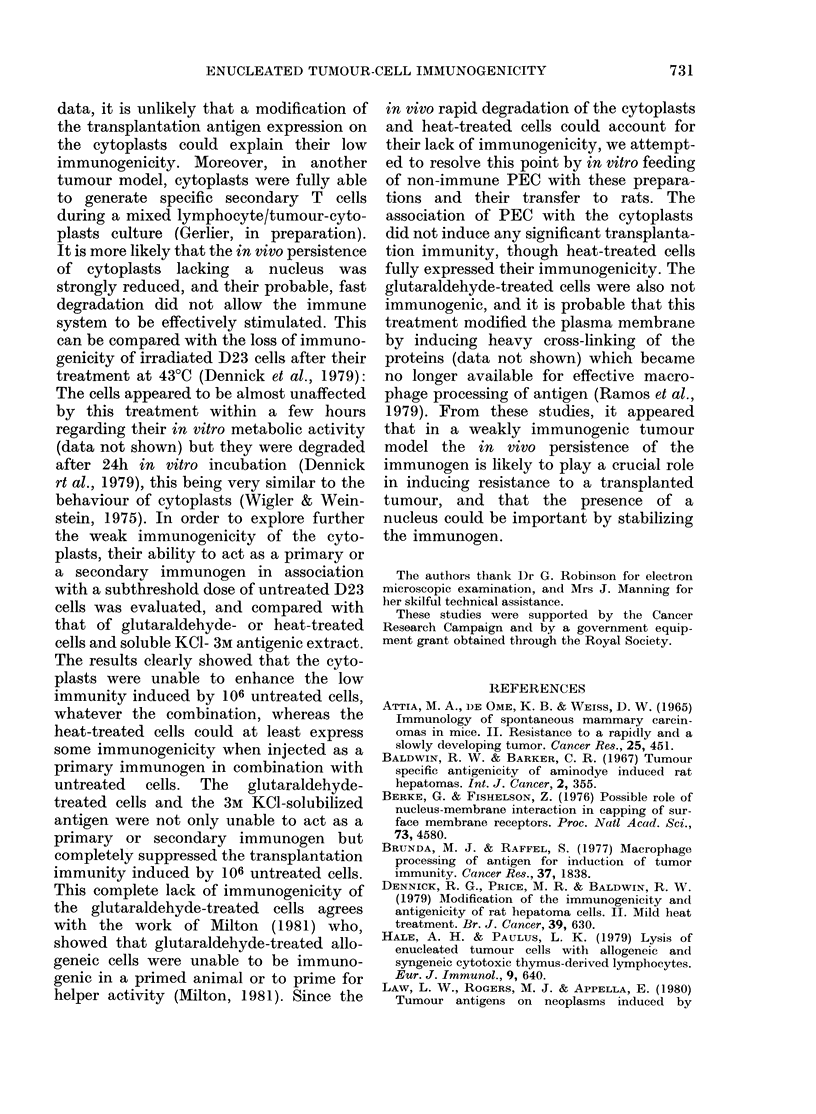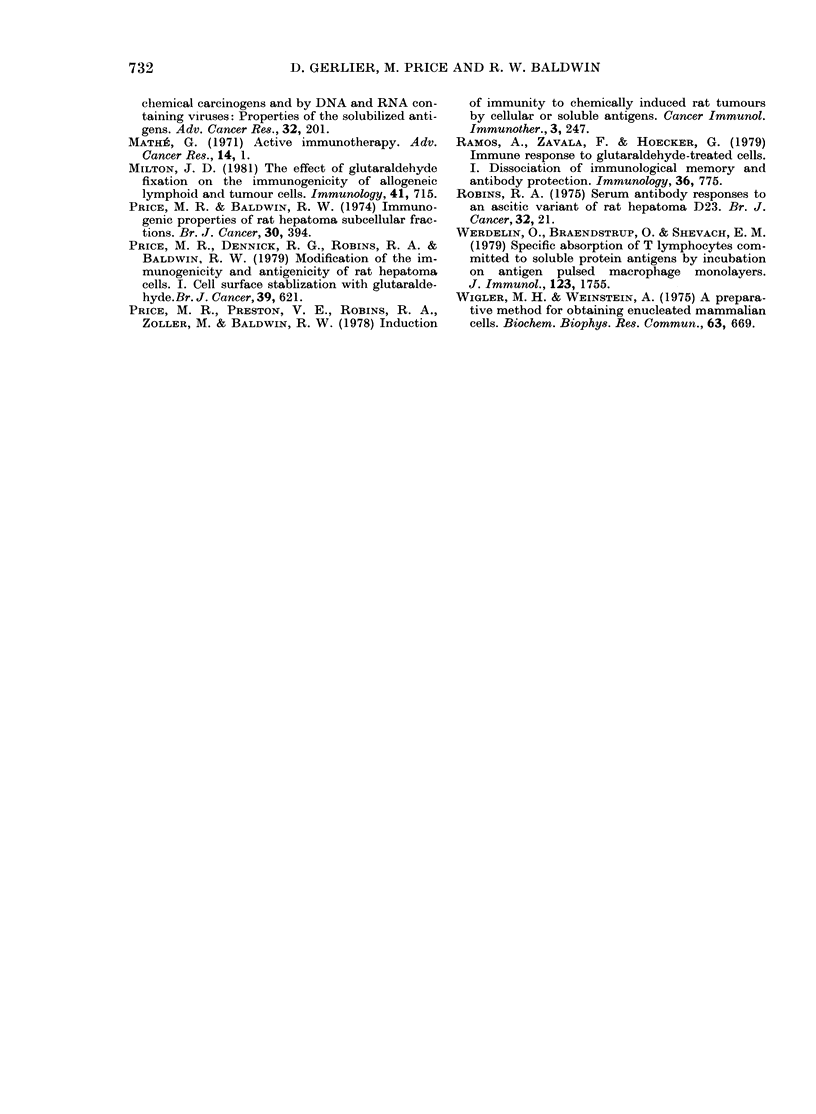# Non-immunogenicity of enucleated rat hepatoma cells in syngeneic animals.

**DOI:** 10.1038/bjc.1981.259

**Published:** 1981-11

**Authors:** D. Gerlier, M. Price, R. W. Baldwin

## Abstract

Cytoplasts and karyoplasts were obtained by ultracentrifugation of Hepatoma D23 cells on a Ficoll gradient containing cytochalasin B. Their nuclear and protein content and their metabolic activity were determined. Three i.p. injections of 2.3 x 10(7) cytoplasts were unable to protect syngeneic WAB/Not rats against an s.c. challenge of 10(4) D23 cells, whereas a similar amount of karyoplasts, or 3 injections of 10(6) irradiated D23 cells, were fully protective. Ability of cytoplasts to act as primary or secondary immunogen were also studied, and compared to that of 0.01% glutaraldehyde-treated cells, 43 degrees C heat-treated cells and 3M KCl-soluble extracts, these preparations also being of weak immunogenicity. Only heat-treated cells behaved as a primary immunogen, whereas none of the preparations provided a secondary stimulation. Moreover, when these preparations were fed in vitro to peritoneal-exudate cells before their injection into rats, cytoplasts and glutaraldehyde-treated cells showed no immunogenicity, whereas heat-treated cells induced full protection against tumour challenge. Therefore, in this tumour model, the in vivo persistence of immunogen and the presence of a nucleus are likely to be crucial in inducing transplantation resistance to tumour.


*Br. J. Cancer* (1981) **44**, 725

### NON-IMMUNOGENICITY OF ENUCLEATED RAT HEPATOMA CELLS IN SYNGENEIC ANIMALS

D. GERLIER*‡, M. PRICE† AND R. W. BALDWIN†

*From the *INSERM U.218, Centre Léon Bérard, 69373 Lyon Cedex 2, France, and the †Cancer Research Campaign Laboratories, University of Nottingham, Nottingham NG7 2RD*

Received 3 July 1981 Accepted 10 August 1981

**Summary.—Cytoplasts and karyoplasts were obtained by ultracentrifugation of Hepatoma D23 cells on a Ficoll gradient containing cytochalasin B. Their nuclear and protein content and their metabolic activity were determined. Three i.p. injections of $2{\cdot}3 \times 10^7$ cytoplasts were unable to protect syngeneic WAB/Not rats against an s.c. challenge of $10^4$ D23 cells, whereas a similar amount of karyoplasts, or 3 injections of $10^6$ irradiated D23 cells, were fully protective. Ability of cytoplasts to act as primary or secondary immunogen was also studied, and compared to that of 0·01% glutaraldehyde-treated cells, 43°C heat-treated cells and 3M KCl-soluble extracts, these preparations also being of weak immunogenicity. Only heat-treated cells behaved as a primary immunogen, whereas none of the preparations provided a secondary stimulation. Moreover, when these preparations were fed *in vitro* to peritoneal-exudate cells before their injection into rats, cytoplasts and glutaraldehyde-treated cells showed no immunogenicity, whereas heat-treated cells induced full protection against tumour challenge. Therefore, in this tumour model, the *in vivo* persistence of immunogen and the presence of a nucleus are likely to be crucial in inducing transplantation resistance to tumour.**

STUDIES ON THE IMMUNOGENICITY of syngeneic tumours have been performed mainly with whole tumour cells, more or less modified by chemical or physical treatment, or with subcellular fractions such as plasma membranes or soluble antigenic extracts (Mathé, 1971). Most of the results have indicated that intact tumour cells inactivated by irradiation or mitomycin C were usually the best immunogen, and that plasma membranes or soluble antigenic extracts were less immunogenic (Law *et al.*, 1980) or non-immunogenic in weakly immunogenic tumour models (Price *et al.*, 1978). Moreover, in the latter study, results obtained on the influence of chemical (glutaraldehyde fixation) or physical treatment (heating) suggested that the degree of immunogenicity of the cell preparation could be related to the level of the residual metabolic activity (Dennick *et al.*, 1979). In order to evaluate this point, we have investigated the immunogenicity of enucleated cell preparations, since it has been demonstrated that such cytoplast and karyoplast preparations retained some degree of metabolic activity (Wigler & Weinstein, 1975) and cell-surface antigen expression (Berke & Fishelson, 1976; Hale & Paulus, 1979).

The mass enucleation procedure was derived from the technique previously described by Wigler & Weinstein (1975). The tumour used in these experiments was the hepatoma D23 initially induced by 4-dimethylaminoazobenzene in a WAB/Not rat. This tumour has been demonstrated to be immunogenic since injections of irradiated viable tumour cells protected the syngeneic rats against a challenge with a low number of cells (Baldwin &

‡ Attaché de Recherche au CNRS, Fellow of the Royal Society (European Science Exchange Programme) from 1.4.1980 to 1.10.1980, to whom requests for reprints should be addressed.

Barker, 1967). The immunogenicity was weak, however, and no transplantation immunity was observed with soluble antigens (Price *et al.*, 1978) or cells treated with 0·01% glutaraldehyde (Price *et al.*, 1979) or heating to 43°C and above (Dennick *et al.*, 1979).

It is reported here that tumour karyoplasts were able to induce transplantation immunity, whereas cytoplasts lacked this property. The ability of the cytoplasts to act as a primary or a secondary immunogen was also tested, and compared with that of glutaraldehyde- or heat-treated cells and with 3M KCl soluble extracts of Hepatoma D23.

### MATERIAL AND METHODS

*Animals*.—Eight- to 10-week-old male WAB/Not rats were used in these experiments.

*Tumour*.—The rat hepatoma D23 (Baldwin & Barker, 1967) and its ascites subline were maintained by s.c. or i.p. transplantation in syngeneic WAB/Not rats (Robins, 1975).

*Enucleation procedure*.—D23 ascites cells were enucleated as described by Wigler & Weinstein (1975), with minor modifications. Briefly, $4 \times 10^7$ D23 cells were washed ×3 in Dulbecco phosphate-buffered saline (DPBS) treated for 10 min in DPBS containing 0·2 g/l EDTA (Sigma Chemical Co., St Louis, Mo) and resuspended in 3 ml of 11% Ficoll (Pharmacia Fine Chemical, Uppsala, Sweden) in Hepes Eagle minimal essential medium HEMEM) (Grand Island Biological Co., Grand Island, N.Y.) containing 10 μg/ml cytochalasin B (Sigma) and carefully layered on a 37°C prewarmed Ficoll gradient consisting of 2 ml each of 25%, 17%, 15%, 13·5% and 12·5% Ficoll in HEMEM containing 10 μg cytochalasin B in 16·5ml MSE ultracentrifuge tubes (MSE, Crawley). A final layer of 2 ml of HEMEM containing 10 μg/ml cytochalasin B was added, and the gradient was ultracentrifuged at 35°C for 90 min at 23,500 rev/min on a 6×16·5ml MSE Aluminium Rotor.

The 0–11% and 11–12·5% interfaces contained enucleated cells (cytoplasts) and the 17–25% interface contained mainly the karyoplasts. Cytoplasts and karyoplasts were washed ×2 in a large excess of medium and incubated 40 min at 37°C to allow them to recover from the cytochalasin B, and finally pelleted at 600 *g* for 5 min before final resuspension. Both fractions were analysed for the number of particles excluding trypan blue, nuclei content (Feulgen staining) and protein content (Lowry). Density of these particles was determined by equilibration on a continuous isotonic Percoll gradient (Pharmacia) made by ultracentrifugation for 20 min at 20,000 *g* on an angular 50 Ti rotor (Beckman Instruments Ltd, Fife) or either 40% or 60% Percoll in HEMEM. The gradients were graduated with coloured beads of known density (Pharmacia). Metabolic activity of the subcellular particles was determined by a short-term incorporation of [$^3$H]-leucine or [$^3$H]dT. $5 \times 10^6$ cells (or particles) were incubated for 1 h at 37°C in 0·5 ml of HEMEM containing 10% foetal calf serum (Grand Island Biological Co.) and 20 μCi of [$^3$H]dT (20–50 mCi/mmol, Radiochemical Centre, Amersham) or in 0·5 ml of leucine-free MEM (Gibco) containing 10% foetal calf serum and 20 μCi of $^3$H-L-leucine (40–60 Ci/mmol, Radiochemical Centre, Amersham). After 3 washes, the cells were disrupted in 1% SDS (Sigma) for 10 min and the DNA and protein were precipitated in 10% TCA (Sigma) for 30 min. After centrifugation the pellet was washed once more with 10% TCA and finally dissolved in 0·5 ml 20g/l $Na_2CO_3$, 0·1N NaOH; 0·1 ml of the solution was added to 1 ml of scintillation liquid and the radioactivity was measured in a Packard liquid scintillation spectrometer. The number of contaminating tumorigenic whole cells in the cytoplast and karyoplast fractions was determined by their ability to induce ascites in WAB/Not rats after i.p. injections of serial dilutions; the results observed (median survival time) was compared with that of rats similarly injected with $10^4$, $10^3$ or $10^2$ untreated D23 cells.

*Tumour-rejection assay*.—Groups of 6 rats were immunized ×3 by weekly i.p. injections with the various cell and subcellular preparations previously irradiated with 150 Gy using a 1000Ci $^{60}$Co source. Rats were challenged 1 week later by s.c. injection of $10^4$ D23 tumour-derived cells prepared by trypsin treatment as previously described (Baldwin & Barker, 1967). To study the ability of various cell preparations (cytoplasts, glutaraldehyde- or heat-treated cells, 3M KCl-soluble extracts) to act as a subthreshold primary or secondary immunogen, the immunization procedure was modified as follows: rats were immunized i.p. either ×2 with one of the test preparations followed by an injection of $10^6$ untreated irradiated D23 cells, or ×1 with $10^6$ untreated irradiated D23 cells followed by 2 injections of the test preparations. Glutaraldehyde (0·01%) and 43°C heat treatment of irradiated D23 cells were performed as previously described (Dennick *et al.*, 1979; Price *et al.*, 1979) and soluble KCl-3M extract of D23 was prepared as previously reported (Price *et al.*, 1978). For the feeding of peritoneal-exudate cells (PEC) with the various cell preparations and their transfer to syngeneic rats, the procedure was as follows: PEC were obtained by aspirating the peritoneal cavity with cold DPBS containing 5 u/ml heparin of rats injected i.p. with autoclaved paraffin oil 4 days previously. The PEC were further washed ×3 at 4°C. $4 \times 10^7$ PEC were incubated with the various cell preparations for 1 h at 37°C in DPBS. Before the i.p. injection in rats, PEC and immunogen were pelleted by 400*g* centrifugation for 5 min and resuspended in DPBS. The rats were challenged s.c. 1 week later with $10^4$ tumour-derived D23 cells. The index for tumour growth is expressed as immunogenicity index as defined by Law *et al.* (1980). This represents the mean tumour volume of the non-immunized control group, divided by the mean tumour volume of immunogen-inoculated rats. The mean tumour volume of each group of rats expressed in $mm^3$ is the arithmetic mean of the tumour volumes of the group. Tumour volume was determined by the formula (Attia *et al.*, 1965) $l \times d^2 \times 0{\cdot}4$, where l is the largest diameter and d the smallest.

### RESULTS

### *Characterization of cytoplasts and karyoplasts (summarized in Table I)*

From $4 \times 10^7$ D23 cells applied on the Ficoll gradient $2{\cdot}8 \times 10^7 \pm 1{\cdot}2$ cytoplasts and $1{\cdot}6 \times 10^7 \pm 0{\cdot}57$ karyoplasts were recovered. More than 95% of the cytoplasts and 60–90% of the karyoplasts retained selective membrane permeability, as determined by their ability to exclude trypan blue. As determined after Feulgen staining, the cytoplasts were contaminated with less than 1% nucleated particles and >90% of the "karyoplasts" were nucleated, which was confirmed by electron microscopy. The cytoplasts consisted of cytoplasmic fragments surrounded by plasma membrane and containing the usual cytoplasmic organelles, and the karyoplast preparation contained mainly nuclei surrounded by a small amount of cytoplasm and plasma membrane. The cytoplasts' metabolic activity was demonstrated by their ability to incorporate 35,330 ct/min of $^3$H-leucine per 0·1 mg of protein in acid precipitate, with a very low DNA synthesis (238 ct/min of [$^3$H]-dT incorporation per 0·1 mg of protein); the karyoplasts incorporated 45,149 ct/min of $^3$H-leucine per 0·1 mg of protein and

TABLE I.—*Yield and characterization of cytoplasts and karyoplasts after enucleation of Hepatoma D23 cells on a Ficoll gradient in the presence of cytochalasin B*

| | Input (whole cells) | Output | |
|---|---|---|---|
| | | Cytoplasts | Karyoplasts |
| No. of cells or particles | $4 \times 10^7$ | $2{\cdot}8 \pm 1{\cdot}2 \times 10^7$ | $1{\cdot}6 \pm 0{\cdot}6 \times 10^7$ |
| Recovery (%) | — | 70 | 40 |
| Trypan-blue dye exclusion (%) | 95 | 99 | 50–95 |
| Nucleated particles (% Feulgen staining) | 100 | < 1 | ⩾ 90 |
| Average density* (g/cm$^3$) | 1·062 | 1·049 | 1·080 |
| Total protein content (mg) | 9·93 ± 2·77 | 0·29 ± 0·13 | 1·98 ± 0·91 |
| Mg of protein/$10^6$ particles | 0·249 ± 0·070 | 0·011 ± 0·004 | 0·132 ± 0·072 |
| Protein synthesis ($^3$H-leu ct/min/0·1mg protein) | 230,730 | 35,330 | 75,651 |
| DNA synthesis ([$^3$H]dT ct/min/0·1mg protein) | 77,530 | 238 | 45,149 |
| No. of Feulgen$^+$ particles/0·1mg protein | | 760–1500 | |
| No. of tumorigenic cells†/0·1mg protein | — | 400–2000 | 5,000–15,500 |
| % of tumorigenic particles | 100 | 0·004–0·02 | 0·7–2·0 |

* Determined by equlibration sedimentation on a continuous Percoll gradient.

† Determined after i.p. induction of ascites and comparison of the median survival time with that of rats similarly injected with $10^4$, $10^3$, or $10^2$ untreated Hepatoma D23 cells.

TABLE II.—*Tumour rejection induced by enucleated Hepatoma D23 cell preparations*

| Immunization* with | Particles/ injection | Protein/ injection (mg) | Tumour incidence | $\chi^2$ test ($P$) | Mean tumour volume† ($mm^3 \pm$s.d.) | Immuno-genicity index‡ | $t$ test ($P$) |
|---|---|---|---|---|---|---|---|
| — | — | — | 12/12 | | $1827 \pm 1379$ | 1 | |
| Whole cells | $10^7$ | 2·5 | 1/18 | < 0·005 | $0·17 \pm 0·75$ | 10,700 | < 0·005 |
| ,, | $10^6$ | 0·25 | 0/12 | < 0·005 | 0 | (∞) | < 0·005 |
| ,, | $10^5$ | 0·025 | 6/12 | < 0·025 | $1104 \pm 1336$ | 1·7 | NS |
| ,, | $10^4$ | 0·0025 | 9/9 | NS | $3151 \pm 1986$ | 0·7 | NS |
| Cytoplasts§ | $2·3 \times 10^7$ | 0·26 | 12/12 | NS | $893 \pm 758$ | 2 | < 0·05 |
| Karyoplasts** | $1·1 \times 10^7$ | 1·9 | 1/12 | < 0·005 | $3·3 \pm 115$ | 55 | < 0·005 |

* WAB/Not rats were given i.p. injections of irradiated antigen preparations ×3 at weekly intervals, followed 1 week later by s.c. challenge with $10^4$ untreated Hepatoma D23 cells.

† Results 21 days after challenge. Tumour volume ($mm^3$) was calculated as $l \times d^2 \times 0·4$, where l = long diameter and d = short diameter (see Attia *et al.*, 1965).

‡ The ratio of mean tumour volume of test rats to mean tumour volume of unimmunized rats (see Law *et al.*, 1980).

§ Number of whole cells per injection $< 5 \times 10^3$.

** Number of whole cells per injection $< 10^5$.

75,651 ct/min of [$^3$H]dT per 0·1 mg of protein, these values being lower than the incorporation by whole D23 cells (230,730 ct/min $^3$H-leucine and 77,530 ct/min [$^3$H]-dT). The contamination of the cytoplast fraction by whole D23 cells was estimated by the *in vivo* tumorigenicity assay to be 400–2000 in 0·1 mg of protein, which was very similar to the number estimated from the [$^3$H]-dT incorporation (calculated as 760–1500 DNA-synthetic particles in 0·1 mg of protein). The karyoplasts were also contained with 500–15,500 tumorigenic cells/0·1 mg protein, corresponding to 1–2% of the nucleated particles.

*Immunogenicity of cytoplasts and karyoplasts*

The results of two separate experiments are detailed in Table II. Three injections of $2·3 \times 10^7$ irradiated cytoplasts (containing 0·26 mg protein) were unable to protect the animals against a challenge of $10^4$ D23 cells (tumour incidence 12/12) but significantly delayed tumour growth (immunogenicity index 2, $0·05 < P < 0·025$), whereas injections of $10^6$ irradiated D23 cells, which were equivalent in protein content (0·25 mg) were fully protective (tumour incidence 0/12). In contrast, immunization with $1·1 \times 10^7$ irradiated karyoplasts protected most of the animals (tumour incidence 1/12). This protection was not related to the contaminating whole cells, since there were $< 10^5$ whole D23 cells per injection and this amount of immunogen was significantly less protective (tumour incidence 6/12, $P < 0·025$) without tumour-growth retardation (immunogenicity index 1·7, $P > 0·10$).

*Ability of cytoplasts to act as a primary immunogen. Comparison with glutaraldehyde- or heat-treated cells and soluble cell extract*

Since cytoplasts alone were only slightly immunogenic, attempts were made to use them as a primary immunogen, and to compare them with other antigenic preparations which have been previously described as weakly or non-immunogenic (Dennick *et al.*, 1979; Price *et al.*, 1978, 1979). The general immunization procedure, which usually gives a consistent protection with $10^6$ irradiated whole cells, was modified as follows: the first 2 injections were performed with the antigen preparation to be tested and followed by one injection of $10^6$ irradiated unmodified D23 cells, before the challenge. The results of a typical experiment are shown (Table III). The use of $2·4 \times 10^7$ cytoplasts in the first 2 immunizations was not significantly protective (tumour incidence 3/6, $P > 0·10$) but delayed the tumour growth (immunogenicity index 4, $P < 0·0125$); this effect was not as good as 3 injections

TABLE III.—*Ability of cell preparations to act as a primary transplantation immunogen*

| 1st and 2nd injection Immunization* with | 3rd injection | Tumour take | $\chi^2$ test ($P$) | Mean tumour volume (mm$^3$ ± s.d.) | Immunogenicity index | $t$ test ($P$) |
|---|---|---|---|---|---|---|
| — | — | 6/6 | | 4537 ± 2413 | 1 | |
| $10^6$ IR cells | $10^6$ IR cells | 2/6 | N.S. | 86 ± 140 | 52 | < 0·0025 |
| — | $10^6$ IR cells | 0/6 | < 0·005 | 0 | (∞) | < 0·0025 |
| $10^7$ 0·01% glutaraldehyde-treated cells | — | 6/6 | N.S. | 3413 ± 2809 | 1·3 | N.S. |
| $10^7$ 0·01% glutaraldehyde-treated cells | $10^6$ IR cells | 5/6 | N.S. | 2713 ± 2123 | 1·7 | N.S. |
| $10^7$ 43°C heat-treated cells | — | 3/6 | N.S. | 796 ± 1297 | 5·7 | < 0·0125 |
| $10^7$ 43°C heat-treated cells | $10^6$ IR cells | 1/6 | < 0·025 | 0·5 ± 1·3 | 8728 | < 0·0025 |
| 2·4 × $10^7$ cytoplasts | $10^6$ IR cells | 3/6 | N.S. | 1137 ± 1474 | 4·0 | < 0·0125 |
| 3M KCl extract (1 mg of protein) | $10^6$ IR cells | 5/6 | N.S. | 3022 ± 2208 | 1·5 | N.S. |

* Schedule as described in Table II.

of $10^6$ cells or even one injection of $10^6$ cells (immunogenicity index 52, $P < 0·0025$ and tumour incidence 0/6). By comparison, 2 immunizations with $10^7$ 0·01% glutaraldehyde-treated cells or 1 mg of 3M KCl soluble-extract protein were unable to induce a protective effect or tumour-growth delay when injected alone, and completely prevented the protective effect of a booster injection of $10^6$ untreated cells (immunogenicity indices 1·7, $P > 0·10$ and 1·5, $P > 0·10$ respectively). In contrast, $10^7$ 43°C heat-treated cells were weakly immunogenic when 2 injections were given (immunogenicity index 5·7, $P < 0·0125$) and one booster injection of $10^6$ irradiated cells greatly increased the immunogenicity of the preparation (tumour incidence 1/6, $P < 0·025$).

*Ability of cytoplasts to act as a secondary immunogen. Comparison with the other antigenic preparations*

The same immunization procedure as described above was used, except that rats first received one injection of $10^6$ irradiated D23 cells, followed by 2 injections of the antigenic preparation to be tested. Table IV shows the results of a representative experiment. The injection of $10^6$ irradiated cells 3 weeks before the challenge was not protective but significantly delayed tumour growth (immunogenicity index 2·4; $0·05 < P < 0·025$), whereas 3 injections were again fully protective (tumour incidence 1/6, $P < 0·025$). Two injections of cytoplasts after administration of $10^6$ irradiated cells did not protect the animals but slightly delayed tumour growth (immunogenicity index 4·3, $P < 0·01$), but not significantly when compared with the effect of the immunization with $10^6$ irradiated cells alone. This absence of modification on the immunogenicity of $10^6$ irradiated cells was also found when 3M KCl extract or 43°C heat-treated cells were used as a secondary immunogen. The use of 0·01% glutaraldehyde-treated cells was again followed by the complete prevention of the weak immunogenicity of $10^6$ irradiated cells (immunogenicity index 1·8, $P > 0·10$).

*Transplantation immunity after the transfer of PEC fed with the various antigen preparations*

Since the *in vitro* incubation of an antigen with macrophage preparations before the injection in the animals has been shown to increase the expression of its immunogenicity (Brunda & Raffel, 1977), we investigated the effect on the immunogenicity of the various antigen preparation after their *in vitro* feeding to PEC. As shown in Table V, the only cell preparation which was fully able to protect the animals against the tumour challenge after their exposure to 4 × $10^7$ PEC was the $10^7$ 43°C heat treated cells (tumour incidence 0/6), $P < 0·005$). The cytoplasts and 0·01% glutaraldehyde-treated cells were ineffective either in protecting or

TABLE IV.—*Ability of cell preparations to act as a secondary transplantation immunogen*

| 1st injection | 2nd and 3rd injection immunization* with | Tumour take | $\chi^2$ test ($P$) | Mean tumour volume ($mm^3 \pm$ s.d.) | Immunogenicity index | $t$ test ($P$) |
|---|---|---|---|---|---|---|
| — | — | 6/6 | | $10{,}950 \pm 5894$ | 1 | |
| $10^6$ IR cells | $10^6$ untreated cells | 1/6 | $< 0{\cdot}025$ | $112 \pm 276$ | 97 | $< 0{\cdot}0025$ |
| $10^6$ IR cells | — | 6/6 | N.S. | $4640 \pm 3983$ | 2·4 | $< 0{\cdot}05$ |
| — | $10^7$ 0·01% glutaraldehyde-treated cells | 6/6 | N.S. | $8900 \pm 2386$ | 1·2 | N.S. |
| $10^6$ IR cells | $10^7$ 0·01% glutaraldehyde-treated cells | 6/6 | N.S. | $6183 \pm 6150$ | 1·8 | N.S. |
| — | $10^7$ 43°C heat-treated cells | 2/6 | N.S. | $1276 \pm 1978$ | 8·6 | $< 0{\cdot}005$ |
| $10^6$ IR cells | $10^7$ 43°C heat-treated cells | 4/6 | N.S. | $3809 \pm 5206$ | 2·9 | $< 0{\cdot}05$ |
| $10^6$ IR cells | $2{\cdot}4 \times 10^7$ cytoplasts | 5/6 | N.S. | $2541 \pm 2977$ | 4·3 | $< 0{\cdot}01$ |
| $10^6$ IR cells | 3M KCl extract (1 mg of protein) | 4/6 | N.S. | $4788 \pm 3886$ | 1·9 | $< 0{\cdot}05$ |

* Immunization schedule as in Table II.

TABLE V.—*Transplantation immunity induced by the transfer of antigen-fed peritoneal-exudate cells (PEC)*

| PEC injected ($4 \times 10^7$) | PEC fed *in vitro* with | Tumour take | $\chi^2$ test ($P$) | Mean tumour volume ($mm^3 \pm$ s.d.) | Immunogenicity index | $t$ test ($P$) |
|---|---|---|---|---|---|---|
| — | — | 6/6 | | $3211 \pm 3300$ | 1 | |
| + | — | 6/6 | N.S. | $4229 \pm 2443$ | 0·76 | N.S. |
| + | $10^6$ IR cells | 1/6 | $< 0{\cdot}025$ | $300 \pm 734$ | 8·9 | $< 0{\cdot}05$ |
| + | $10^7$ 0·01% glutaraldehyde-treated cells | 6/6 | N.S. | $2724 \pm 3093$ | 1·2 | N.S. |
| + | $10^7$ 43°C heat-treated cells | 0/6 | $< 0{\cdot}005$ | 0 | ($\infty$) | $< 0{\cdot}005$ |
| + | $1{\cdot}5 \times 10^7$ cytoplasts | 6/6 | N.S. | $3227 \pm 929$ | 1 | N.S. |

$4 \times 10^7$ PEC were incubated with the irradiated tumour cell preparation for 1 h at 37°C and injected i.p. into rat. One week later rats were challenged with $10^4$ untreated D23 cells.

delaying the tumour growth (immunogenicity index 1; $P > 0{\cdot}40$ and 1·2; $P > 0{\cdot}40$, respectively) though PEC fed with only $10^6$ irradiated cells were fully protective (tumour incidence 1/6, $P < 0{\cdot}025$).

### DISCUSSION

The data obtained in this study demonstrate that cytoplasts and karyoplasts can be isolated after a mass enucleation of D23 hepatoma cells on a Ficoll gradient in the presence of cytochalasin B; these preparations retain partial metabolic activity, in accordance with previous findings (Wigler & Weinstein, 1975). The karyoplasts were demonstrated to be immunogenic in inducing a syngeneic transplantation tumour resistance. However, karyoplasts are only about one tenth as effective as whole irradiated cells, as determined by the amount of material required for full protection, and their immunogenicity is likely to be due to the presence of residual cytoplasm and plasma membranes, since naked nuclei have been previously shown to be non-immunogenic (Price & Baldwin, 1974). In contrast, cytoplasts induced slight delay of the tumour growth. The low immunogenicity could not be related to the Ficoll and cytochalasin B treatment *per se*, since a sham treatment of cells did not affect their immunogenicity (data not shown), nor to the reduced amount of material used to immunize the animals, since the amount of protein injected was similar to that of $10^6$ D23 tumour cells, which were significantly protective. Moreover, as previously observed (Berke & Fishelson, 1976; Hale & Paulus, 1979), the cytoplasts (and karyoplasts) fully expressed the rat major histocompatibility-complex antigens and the specific serologically defined D23 antigen (Holmes, personal communication). Although we have no quantitative data, it is unlikely that a modification of the transplantation antigen expression on the cytoplasts could explain their low immunogenicity. Moreover, in another tumour model, cytoplasts were fully able to generate specific secondary T cells during a mixed lymphocyte/tumour-cytoplasts culture (Gerlier, in preparation). It is more likely that the *in vivo* persistence of cytoplasts lacking a nucleus was strongly reduced, and their probable, fast degradation did not allow the immune system to be effectively stimulated. This can be compared with the loss of immunogenicity of irradiated D23 cells after their treatment at 43°C (Dennick *et al.*, 1979): The cells appeared to be almost unaffected by this treatment within a few hours regarding their *in vitro* metabolic activity (data not shown) but they were degraded after 24h *in vitro* incubation (Dennick *rt al.*, 1979), this being very similar to the behaviour of cytoplasts (Wigler & Weinstein, 1975). In order to explore further the weak immunogenicity of the cytoplasts, their ability to act as a primary or a secondary immunogen in association with a subthreshold dose of untreated D23 cells was evaluated, and compared with that of glutaraldehyde- or heat-treated cells and soluble KCl- 3M antigenic extract. The results clearly showed that the cytoplasts were unable to enhance the low immunity induced by $10^6$ untreated cells, whatever the combination, whereas the heat-treated cells could at least express some immunogenicity when injected as a primary immunogen in combination with untreated cells. The glutaraldehyde-treated cells and the 3M KCl-solubilized antigen were not only unable to act as a primary or secondary immunogen but completely suppressed the transplantation immunity induced by $10^6$ untreated cells. This complete lack of immunogenicity of the glutaraldehyde-treated cells agrees with the work of Milton (1981) who, showed that glutaraldehyde-treated allogeneic cells were unable to be immunogenic in a primed animal or to prime for helper activity (Milton, 1981). Since the *in vivo* rapid degradation of the cytoplasts and heat-treated cells could account for their lack of immunogenicity, we attempted to resolve this point by *in vitro* feeding of non-immune PEC with these preparations and their transfer to rats. The association of PEC with the cytoplasts did not induce any significant transplantation immunity, though heat-treated cells fully expressed their immunogenicity. The glutaraldehyde-treated cells were also not immunogenic, and it is probable that this treatment modified the plasma membrane by inducing heavy cross-linking of the proteins (data not shown) which became no longer available for effective macrophage processing of antigen (Ramos *et al.*, 1979). From these studies, it appeared that in a weakly immunogenic tumour model the *in vivo* persistence of the immunogen is likely to play a crucial role in inducing resistance to a transplanted tumour, and that the presence of a nucleus could be important by stabilizing the immunogen.

The authors thank Dr G. Robinson for electron microscopic examination, and Mrs J. Manning for her skilful technical assistance.

These studies were supported by the Cancer Research Campaign and by a government equipment grant obtained through the Royal Society.

REFERENCES

ATTIA, M. A., DE OME, K. B. & WEISS, D. W. (1965) Immunology of spontaneous mammary carcinomas in mice. II. Resistance to a rapidly and a slowly developing tumor. *Cancer Res.*, **25**, 451.

BALDWIN, R. W. & BARKER, C. R. (1967) Tumour specific antigenicity of aminodye induced rat hepatomas. *Int. J. Cancer*, **2**, 355.

BERKE, G. & FISHELSON, Z. (1976) Possible role of nucleus-membrane interaction in capping of surface membrane receptors. *Proc. Natl Acad. Sci.*, **73**, 4580.

BRUNDA, M. J. & RAFFEL, S. (1977) Macrophage processing of antigen for induction of tumor immunity. *Cancer Res.*, **37**, 1838.

DENNICK, R. G., PRICE, M. R. & BALDWIN, R. W. (1979) Modification of the immunogenicity and antigenicity of rat hepatoma cells. II. Mild heat treatment. *Br. J. Cancer*, **39**, 630.

HALE, A. H. & PAULUS, L. K. (1979) Lysis of enucleated tumour cells with allogeneic and syngeneic cytotoxic thymus-derived lymphocytes. *Eur. J. Immunol.*, **9**, 640.

LAW, L. W., ROGERS, M. J. & APPELLA, E. (1980) Tumour antigens on neoplasms induced by chemical carcinogens and by DNA and RNA containing viruses: Properties of the solubilized antigens. *Adv. Cancer Res.*, **32**, 201.

MATHÉ, G. (1971) Active immunotherapy. *Adv. Cancer Res.*, **14**, 1.

MILTON, J. D. (1981) The effect of glutaraldehyde fixation on the immunogenicity of allogeneic lymphoid and tumour cells. *Immunology*, **41**, 715.

PRICE, M. R. & BALDWIN, R. W. (1974) Immunogenic properties of rat hepatoma subcellular fractions. *Br. J. Cancer*, **30**, 394.

PRICE, M. R., DENNICK, R. G., ROBINS, R. A. & BALDWIN, R. W. (1979) Modification of the immunogenicity and antigenicity of rat hepatoma cells. I. Cell surface stablization with glutaraldehyde. *Br. J. Cancer*, **39**, 621.

PRICE, M. R., PRESTON, V. E., ROBINS, R. A., ZOLLER, M. & BALDWIN, R. W. (1978) Induction of immunity to chemically induced rat tumours by cellular or soluble antigens. *Cancer Immunol. Immunother.*, **3**, 247.

RAMOS, A., ZAVALA, F. & HOECKER, G. (1979) Immune response to glutaraldehyde-treated cells. I. Dissociation of immunological memory and antibody protection. *Immunology*, **36**, 775.

ROBINS, R. A. (1975) Serum antibody responses to an ascitic variant of rat hepatoma D23. *Br. J. Cancer*, **32**, 21.

WERDELIN, O., BRAENDSTRUP, O. & SHEVACH, E. M. (1979) Specific absorption of T lymphocytes committed to soluble protein antigens by incubation on antigen pulsed macrophage monolayers. *J. Immunol.*, **123**, 1755.

WIGLER, M. H. & WEINSTEIN, A. (1975) A preparative method for obtaining enucleated mammalian cells. *Biochem. Biophys. Res. Commun.*, **63**, 669.